# Amino Acid Substitutions and Differential Gene Expression of Outer Membrane Proteins in Adherent-Invasive *Escherichia coli*

**DOI:** 10.3389/fmicb.2019.01707

**Published:** 2019-08-06

**Authors:** Carla Camprubí-Font, Belén Ruiz del Castillo, Silvia Barrabés, Luis Martínez-Martínez, Margarita Martinez-Medina

**Affiliations:** ^1^Laboratory of Molecular Microbiology, Department of Biology, Universitat de Girona, Girona, Spain; ^2^Service of Microbiology, University Hospital Marques de Valdecilla-Valdecilla Biomedical Research Institute (IDIVAL), Santander, Spain; ^3^Biochemistry and Molecular Biology Unit, Department of Biology, Universitat de Girona, Girona, Spain; ^4^Microbiology Unit, University Hospital Reina Sofia, Córdoba, Spain; ^5^Department of Microbiology, University of Córdoba, Córdoba, Spain; ^6^Instituto Maimónides de Investigación Biomédica de Córdoba (IMIBIC), Córdoba, Spain

**Keywords:** adherent-invasive *E. coli*, virulence genes, amino acid substitutions, gene expression, outer membrane protein, infection

## Abstract

Variations in the sequence and/or the expression of outer membrane proteins (OMPs) may modulate bacterial virulence. OmpA and OmpC have been involved in the interaction of adherent-invasive *Escherichia coli* (AIEC) strain LF82 with intestinal epithelial cells (IECs). Scarce data exist about OMPs sequence variants in a collection of AIEC strains, and no study of OMPs expression during infection exists. We aimed to determine whether particular mutations or differential expression of OMPs are associated with AIEC virulence. The *ompA, ompC*, and *ompF* genes in 14 AIEC and 30 non-AIEC strains were sequenced by Sanger method, and the protein expression profile was analyzed by urea-SDS-PAGE. Gene expression was determined during *in vitro* bacterial infection of intestine-407 cells by RT-qPCR. The distribution of amino acid substitutions in OmpA-A200V, OmpC-S89N, V220I, and W231D associated with pathotype and specific changes (OmpA-A200V, OmpC-V220I, D232A, OmpF-E51V, and M60K) correlated with adhesion and/or invasion indices but no particular variants were found specific of AIEC. OMPs protein levels did not differ according to pathotype when growing in Mueller-Hinton broth. Interestingly, higher OMPs gene expression levels were reported in non-AIEC growing in association with cells compared with those non-AIEC strains growing in the supernatants of infected cultures (*p* < 0.028), whereas in AIEC strains *ompA* expression was the only increased when growing in association with cells (*p* = 0.032), but they did not significantly alter *ompC* and *ompF* expression under this condition (*p* > 0.146). Despite no particular OMPs sequence variants have been found as a common and distinctive trait in AIEC, some mutations could facilitate a better interaction with the host. Moreover, the different behavior between pathotypes regarding OMPs gene expression at different stages of infection could be related with the virulence of the strains.

## Introduction

Microbiota alterations have risen as putative contributors toward the development of Crohn’s disease (CD), a chronic condition that may affect the entire gastrointestinal tract (Sartor, 2006; Kaser et al., 2010). Increased abundance of *Proteobacteria* together with a decrease in *Firmicutes* has been frequently identified in the gut of CD patients (Darfeuille-Michaud et al., 2004; Martinez-Medina et al., 2009). Among the proinflammatory bacteria found to be increased in CD patients, colonization by the adherent-invasive *E. coli* (AIEC) pathotype has been revealed (Boudeau et al., 1999; Darfeuille-Michaud et al., 2004; Martinez-Medina et al., 2009). This pathotype is characterized by its ability to adhere to and invade intestinal epithelial cells (IECs), as well as to replicate and survive inside macrophages without triggering host-cell death. AIEC identification is currently performed by highly time-consuming *in vitro* cell-line assays, which are laborious and non-standardizable due to the absence of specific genetic signatures for AIEC strains.

The mechanisms and virulence factors by which this pathotype is able to colonize and translocate the inflamed intestinal epithelium have been extensively studied (for review see Palmela et al., 2018). In most cases, the key pathogenic determinants are surface structures, such as the Type 1 pili adhesin, ChiA, and LpfA. These proteins are involved in the bacterial adhesion to and translocation of IECs via the interaction with CEACAM6, CHI3L1, and GP2 receptors, which are overexpressed in CD patients (Boudeau et al., 2001; Carvalho et al., 2009; Chassaing et al., 2011; Low et al., 2013). Some outer membrane proteins (OMPs) act in concert with these virulence genes to enhance AIEC pathogenicity. This is the case for the outer membrane protein OmpA, in which five amino acid variants have been suggested to favor bacterial invasion (Rolhion et al., 2010). OmpA is present in outer membrane vesicles and improves bacterial invasion by binding the chaperone Gp96 which is more frequently expressed on the apical surface of the epithelium in ileal CD patients (Rolhion et al., 2010). Additionally, it has been shown that OmpC has an indirect role in the AIEC phenotype, as adhesion and invasion indices are reduced when *ompC* expression is decreased by the σ^*E*^ regulatory pathway (Rolhion et al., 2007). However, in both cases only one AIEC strain was assessed (LF82) (Rolhion et al., 2007, 2010). Therefore, due to the high genetic diversity of the AIEC pathotype and its similarity with extraintestinal pathogenic *E. coli* (ExPEC) and commensal strains (Baumgart et al., 2007; Martinez-Medina et al., 2009; Miquel et al., 2010; Nash et al., 2010), examination of OmpA amino acid substitutions or *ompC* gene expression across a larger collection of AIEC/non-AIEC strains should be completed. Finally, no studies regarding the OmpF and AIEC phenotype have been carried out, yet its high similarity with OmpC make it susceptible to be examined.

AIEC invasion of IECs occurs by the formation of endocytic vesicles in a macropinocytosis-like mechanism mediated by host cell cytoskeleton reorganization (Boudeau et al., 1999). Internalized bacteria are mainly located in late endosomes, but some can also be found free in the host cytoplasm (Wine et al., 2009). Within the endosomal pathway, bacteria must be resistant to acidic pH, oxidative stress and proteolytic activity. OmpC and OmpF expression can be modulated by pH and osmolarity in *E. coli* (Sato et al., 2000; Sainz et al., 2005; Rolhion et al., 2007), including the AIEC LF82 strain (Rolhion et al., 2007). Thus, one may suspect that OMPs expression regulation is a mechanism by which AIEC can adapt to intracellular IECs persistence and facilitate adaptation to changing environmental conditions of a CD intestine. To date, AIEC gene expression has been scarcely studied (Bringer et al., 2005; Zhang et al., 2015) and there is no literature focused on differential gene expression during AIEC infection.

This work aims to identify gene mutational and differential expression patterns in OMPs among a collection of AIEC and non-AIEC strains isolated from the human intestine, to determine if there is a common and distinctive trait among AIEC strains that could be linked with their virulence and to evaluate OMPs expression levels during the AIEC infection process.

## Materials and Methods

### Bacterial Strains

A collection of 13 AIEC strains isolated from CD patients and controls in a previous study (Martinez-Medina et al., 2009) was analyzed together with 30 non-AIEC strains that were isolated from the same group of subjects but that did not present the adherent-invasive phenotype (Supplementary Table S1). This study was conducted under the approval ofthe Ethics Committee of Clinical Investigation of the Hospital Josep Trueta of Girona on May 22, 2006. All subjects gave written informed consent in accordance with the Declaration of Helsinki. The AIEC LF82 strain was included as reference (Boudeau et al., 1999). Adhesion and invasion indices were previously assessed (Martinez-Medina et al., 2009; Camprubí-Font et al., 2018) following gentamicin protection assays.

### Amplification and Gene Sequencing

Strains were grown in Luria-Bertani (LB) broth overnight at 37°C. Total DNA was extracted by NucleoSpin^®^ Tissue (Macherey-Nagel GmbH & Co., KG) kit by following the manufacturer’s instructions. All genes (*ompA, ompC*, and *ompF*) were amplified in a PCR reaction containing 1x Buffer II, 2 mM of MgCl_2_, 0.2 mM of dNTPs, 0.5 mM of the corresponding primers (Supplementary Table S2), (Huijsdens et al., 2002; Viveiros et al., 2007; Ruiz del Castillo et al., 2013) 1 U/reaction of AmpliTaq Gold polymerase (Thermo Fisher Scientific, United States) and 1 μL of DNA Template at 20 ng/μL in a final volume of 20 μL. The amplification PCR program consisted of 1 cycle at 95°C for 10 min, 35 cycles of 45 s at 95°C, 45 s at the primer annealing temperature (Supplementary Table S2) and 1 min at 72°C, and one cycle at 72°C for 10 min. The presence of only one band was checked by running the product on a 1.5% agarose gel. PCR products were cleaned by ExoSap (Thermo Fisher Scientific, United States) and sequenced with the Sanger method in both directions using the same primers as stated for amplification by Macrogen service (Korea). Consensus sequences were deposited under the accession number MH754762 – MH754812 (*ompA*), MH754813 – MH754863 (*ompC*), and MH754864 – MH754913 (*ompF*) in the GenBank database.

### Sequence Analysis

The consensus sequence for each strain was aligned with the corresponding gene sequence of the LF82 reference strain extracted from the database (Accession id: CU651637.1) using BioEdit (Hall, 1999). To identify non-synonymous point mutations, DNA sequences were translated to amino acids using EMBOSS Transeq (EMBL-EBI) (Rice et al., 2000).

Phylogenetic analyses were represented with an amino acid-based reticular tree constructed with Popart software (version 1.7) using the median-joining algorithm for each gene. In all cases, gene sequences from other AIEC, commensal, ExPEC (UPEC, MNEC, and APEC) and IPEC (EAEC, EHEC, ETEC, EIEC, DAEC, and STEC) strains retrieved from GenBank were also included: AIEC UM146 strain (CP002167.1); AIEC NRG857c strain (CP001855.1); Commensal HS strain (CP000802.1); Commensal K-12 strain (CP012868.1); Commensal ED1a strain (CU928162.2); UPEC CFT073 strain (NC_004431.1); UPEC 536 strain (CP000247.1); UPEC UMN026 strain (CU928161.2); MNEC S88 strain (CU928163.2); APEC APEC01 strain (CP000468.1); EAEC 042 strain (NC_017626.1); EHEC EDL933 strain (AE005174.2); EHEC 0154 Sakai strain (BA000007.2); ETEC E24377A strain (CP000800.1); EIEC CFSAN0299787 strain (CP011416.1); DAEC SaT040 strain (CP014495.1); STEC ST540 strain (CP007265.1).

Protein crystal structures were obtained from the RCSB Protein Data Bank (PDB) data using as a reference (OmpA 1G90, OmpC 2J1N, and OmpF 2OMF) and modified with Pymol.

### OMPs Isolation and Separation by SDS-PAGE

OMPs were isolated as described previously (Hernández-Allés et al., 1999) with some modifications. After overnight culture in Mueller-Hinton (MH) broth, each strain was harvested by centrifugation and resuspended in 1 mL Tris-Mg buffer (10 mM Tris-HCl, 5 mM MgCl2, pH 7.3). Cells were sonicated at 15% amplitude using a 1/8” diameter tapered horn for 5 cycles as previously described. Unbroken cells were eliminated by centrifugation at 5000 rpm and 4°C for 5 min, and cell envelopes were recovered by centrifugation at 17,000 rpm and 4°C for 30 min. Membranes were solubilized in 2% sodium lauroyl sarcosinate for 30 min at room temperature, and centrifuged at 17,000 rpm and 4°C for 30 min. The pellet was washed in 1 mL Tris-Mg buffer, centrifuged as above and finally solubilized in 40 μL Tris-Mg buffer. Protein concentration was quantified with the Quick Start Bradford 1xDye Reagent (BioRad, United States).

Separation analysis of OMPs was performed in urea-SDS-PAGE. The resolving gel was 10% acrylamide-0.27% *N,N’*-Methylenebisacrylamide, 6 M Urea, 375 mM Tris-HCl pH 8.8, 0.2% SDS, 0.2% TEMED, and 0.075% amonium persulfate. The stacking gel was 5% acrylamide-0.13% *N,N’*-Methylenebisacrylamide, 6 M Urea, 125 mM Tris-HCl pH6.8, 0.2% SDS, 0.06% TEMED, and 0.1% amonium persulfate. A total of 15 μg of protein was loaded, and the gel was stained with Coomassie as in Hernández-Allés et al. (1999).

### Infection and RNA Extraction

Intestinal epithelial cells (I-407 cell line; ATCC CCL-6) were grown in a T25 flask to a density of 6.6 × 10^6^ total IECs at 37°C with 5% CO_2_ for 20 h. Each flask was infected at a multiplicity of infection (MOI) of 100 without centrifugation. After 4 h, the supernatant containing bacteria growing in suspension (SN) was separated from the infected cells (A/I) which contained the adherent and invasive bacteria. The monolayer was washed twice with EMEM (Lonza, Switzerland) + 10% inactivated FBS (Gibco, United States), and cells were collected with a scraper. Then, both fractions (SN and A/I) were centrifuged at 3000 × *g* for 10 min at 4°C. The supernatant of each fraction was discarded, and the pellet was washed with PBS (Lonza, Switzerland). Total RNA extraction was performed with the TRIzol Max Bacterial Isolation kit (Invitrogen, United States) with some modifications. After the addition of chloroform incubation at room temperature for 15 min was performed, and an overnight precipitation at −20°C was performed after isopropanol addition. Subsequently, a DNase I- RNase-free treatment (Thermo Fisher Scientific, United States) was used to eliminate any possible DNA contamination in the sample.

### Gene Expression Quantification by RT-qPCR

Total RNA (2 μg) was reverse transcribed using random hexamer primers with a High-Capacity cDNA Reverse Transcription Kit with RNase inhibitor (Thermo Fisher Scientific, United States). RT-qPCR was performed with primers (Supplementary Table S2) designed using Primer3 (version 0.4.0) based on the gene sequences of the strain collection obtained in this study. All primers were further analyzed with NetPrimer to select the optimal primer pair. The amplification reactions were carried out in a total volume of 20 μL containing: 10 μL of Power SYBR^TM^ Green PCR Master Mix 2x (Applied Biosystems, Foster City, CA, United States), 300 nM of each primer and RNase free water up to the final volume. All quantitative PCRs were performed using a 7500 Real-Time PCR system (Applied Biosystems, Foster City, CA, United States). Thermal cycling conditions consisted of an initial step at 50°C for 30 min, a PCR activation step at 95°C for 10 min to denature DNA and activate Ampli-Taq Gold polymerase, and a further denaturation step of 40 cycles (95°C for 15 s) followed by an annealing and extension step at 60°C for 1 min. Data were collected and analyzed with the 7500 SDS system software version 1.4 (Applied Biosystems, Foster City, CA, United States).

Samples were quantified in triplicate. The relative transcripts abundance (RTA) for each gene of interest was determined by applying the comparative threshold cycle (Ct) method (Pfaffl, 2001). Differences in expression levels were normalized against the *E. coli* housekeeping gene *gapA*, which was measured in the same sample (ΔCt) and compared with LF82 A/I gene expression by the equation RTA = Efficiency ^(Ct target gene reference strain – Ct target gene sample)/Efficiency ^(Ct constitutive gene reference strain – Ct constitutive gene sample) (Pfaffl, 2001). The efficiency was calculated based on the standard curve of each set of primers (E = 10^–1/slope^). A log10 RTA value similar to 0 indicates a similar expression level between the corresponding sample and the reference strain (LF82 A/I). Additionally, the 16S rRNA copy number was also evaluated in all samples in duplicate. Reactions were carried out in a total volume of 20 μL, and the reaction contained a 1x Taqman universal PCR master mixture (Applied Biosystems, Foster City, CA, United States), 300 nM of each *E. coli*-specific oligonucleotide primer and 100 nM fluorescence-labeled *E. coli*-specific probe (Supplementary Table S2). The same thermal cycle as explained above was performed.

### Statistical Analysis

Differences in the amino acid present in each variable position between pathotype and phylogroup origin, were calculated using the X^2^-test. For phylogroup analysis, the atypical strain was not contemplated. To compare the mean adhesion and invasion indices (previously assessed in Martinez-Medina et al., 2009; Camprubí-Font et al., 2018) between more than two amino acid variants the non-parametric Kruskal–Wallis test was used, while the Mann–Whitney *U*-test was performed to analyze pairwise comparisons. Differences in OMPs protein expression and profiles were determined using the X^2^-test. To analyze differential gene expression according to pathotype, the Mann–Whitney *U*-test for two independent samples was applied. Gene expression versus adhesion and invasion capacities was calculated using Spearman’s correlation. Finally, paired analysis according to the condition was performed with the Wilcoxon signed-rank test. A *p* ≤ 0.05 was considered statistically significant in all cases.

## Results

### OMPs Sequence Variants

**FIGURE 1:**
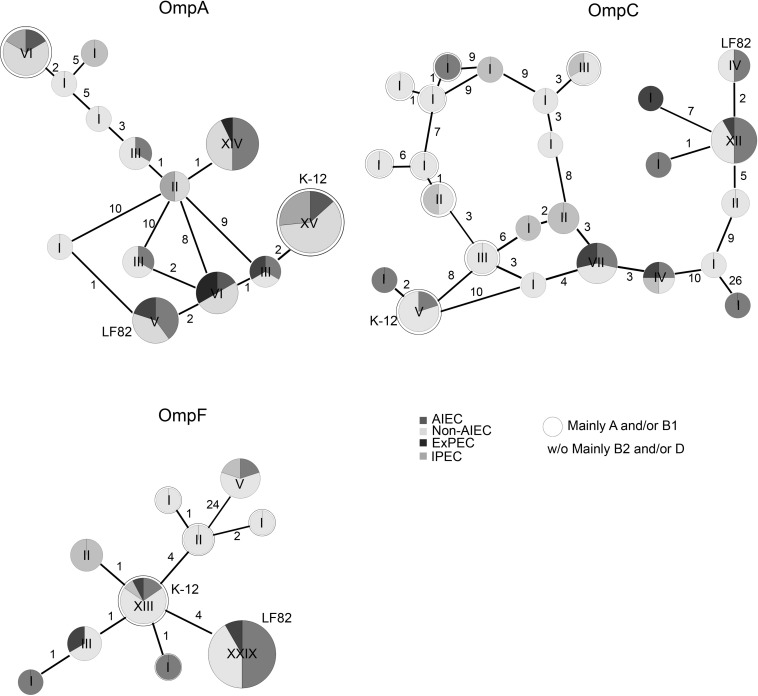
Reticulated trees representing the distribution of strains carrying specific amino acid substitutions in OMPs. The number of strains in each variant is pointed out by Roman numerals. The number of amino acid changes between each variant is indicated. The LF82 strain was used as a reference. ExPEC and IPEC gene sequences were retrieved from NCBI. The rounded circles are groups of strains mainly from the A and/or B1 phylogroups while the others (without circles or w/o) mainly involve B2 and/or D-phylogroup strains.

Changes in the sequence of *ompA, ompC*, and *ompF* genes in 43 *E. coli* isolates were studied by PCR and subsequent Sanger sequencing and compared to the OMPs gene sequence of the LF82 strain. One hundred percent amplification was achieved for the *ompA* and *ompF* genes while for the *ompC* gene was 97.6% (one non-AIEC was PCR-negative). Of the three OMPs, OmpC protein was the most variable (81% similarity), and OmpA was the most conserved (94% similarity) among all of the strains. The similarity for OmpF was 91%. OMPs sequences of our strain collection were studied together with the OMPs sequences of other pathotypes, which were retrieved from the genbank (61 total OmpA sequences, 58 total OmpC sequences and 58 total OmpF sequences). The sequence of OmpA in all strains varied at 17 amino acid positions and was grouped in 13 variants (Figure 1 and Supplementary Table S3). OmpC and OmpF sequences differed at 65 and 30 positions resulting in 25 and 10 variants, respectively (Figure 1 and Supplementary Tables S4, S5).

Once the gene sequences of our strain collection (*n* = 44) were compared with the ones retrieved from databases (*n* = 17), the most common variant in OmpA was present in 15/61 strains, which shared the same OmpA sequence as K-12 (Supplementary Table S3). The non-AIEC strains were predominant in this OmpA variant (*n* = 9); nonetheless, some AIEC (*n* = 2) and IPEC (*n* = 4) strains also harbored the variant. The second most common variant (*n* = 14) was found in AIEC (*n* = 7), ExPEC (*n* = 1), and non-AIEC (*n* = 6) strains. Moreover, the LF82 OmpA variant was present in two AIEC strains, two non-AIEC strains and one ExPEC. For OmpC, the most common variant (12/58 strains) varied only in two positions in comparison with the LF82 sequence and comprised 6 AIEC, 5 non-AIEC and 1 ExPEC strains (Supplementary Table S4). Again, the LF82 variant was shared with one AIEC and two non-AIEC strains. Finally, the OmpF protein presented the lowest number of variants, although the number of variable positions was higher than that of OmpA. In this case, 29/58 strains, including 11 AIEC, 14 non-AIEC, and 4 ExPEC strains, displayed the same amino acid sequence as the LF82 strain (Supplementary Table S5). The second most common variant comprised 13/58 strains: 2 AIEC, 9 non-AIEC, 1 IPEC, and 1 ExPEC in which K-12 was included.

Overall, no protein variants were specifically associated with AIEC strains, and OMPs LF82 variants were also detected in non-AIEC strains.

### Distribution of Amino Acid Substitutions in OMPs

**FIGURE 2:**
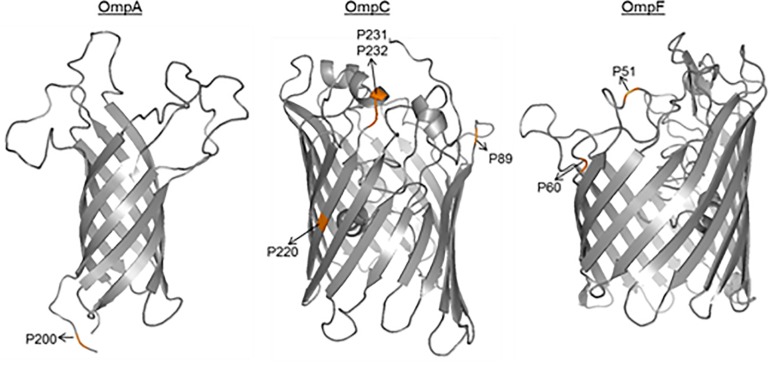
3D- monomer representations of the transmembrane domains of OMPs. The amino acid positions related to pathotype and/or adhesion and invasion indices are highlighted.

Although no particular sequence variants were associated with the AIEC pathotype, the differential distribution of point mutations was analyzed. In our strain collection, similar amino acid distribution between AIEC and non-AIEC were reported for the OmpA mutations previously detected between LF82 and K-12 strains (V114I, F131V, D132Y, T228N, and A276G) (Rolhion et al., 2010; *p* > 0.101; Supplementary Figure S1). In contrast, in this study, the valine (V) residue in position 200 of OmpA sequence was more frequently found in AIEC than in non-AIEC strains, and three OmpC positions (S89N, V220I, and W231D) were also differentially distributed among AIEC and non-AIEC strains (Table 1, Supplementary Table S6, and Figure 2). Increased adhesion indices correlated with V residue in the periplasmic position 200 of OmpA (*p* = 0.044), and increased invasion indices with V in the extracellular position 220 of OmpC (*p* < 0.022) (Table 1 and Figure 2). Additional amino acid substitutions correlated with higher adhesion (OmpC extracellular position 232; OmpF extracellular position 51; OmpF extracellular position 60) and invasion (OmpC extracellular positions 220 and 232) indices (*p* < 0.040; Table 1 and Figure 2).

**TABLE 1:** Variable positions in OMPs sequence related to pathotype or AIEC phenotypic characteristics are depicted.

		**All strains**			**Adhesion index**		**Invasion index**	
**Variable position**	**Amino acid**	**Non-AIEC (*n* = 30)**	**AIEC (*n* = 14)**	***p*-value**	**Bacteria/cell**	***p*-value**	% ^*^	***p*-value**
**OmpA**								
200	A	80.0%	42.9%	**0.018**	4.1 ± 7.4	**0.044**	0.184 ± 0.457	0.084
	V	20.0%	57.1%		7.8 ± 7.9		0.224 ± 0.359	
**OmpC**								
232	D	62.1%	85.7%	0.108	6.5 ± 8.0	**0.017**	0.229 ± 0.474	**0.002**
	A	37.9%	14.3%		2.9 ± 6.5		0.060 ± 0.157	
231	W	31.0%	64.3%	**0.041**	6.4 ± 8.1	0.189	0.315 ± 0.589	0.058
	D	69.0%	35.7%		4.6 ± 7.5		0.079 ± 0.158	
89	S	31.0%	64.3%	**0.041**	6.4 ± 8.1	0.372	0.314 ± 0.589	0.109
	N	69.0%	35.7%		4.7 ± 7.4		0.080 ±0.158	
220	V	20.7%	57.1%	**0.022**	7.9 ± 8.6	0.078	0.366 ±0.653	0.022
	I	79.3%	42.9%		4.2 ± 7.0		0.087 ± 0.169	
**OmpF**								
51	E	80.0%	100.0%	0.084	6.1 ± 7.9	**0.040**	0.198 ± 0.433	0.322
	V	20.0%	0.0%		0.2 ± 0.4		0.021 ± 0.019	
60	M	80.0%	100.0%	0.084	6.1 ± 7.9	**0.040**	0.198 ± 0.433	0.322
	K	20.0%	0.0%		0.2 ± 0.4		0.021 ± 0.019	

*In bold statistically significant comparisons are indicated. For each position, the first amino acid presented is the one present in the LF82 reference strain. Adhesion and invasion indices were obtained from raw data in Supplementary Table S1*. ^*^Percentage of intracellular bacteria after 1 h gentamicin treatment relative to the inoculum.

A number of variant positions were associated with the phylogenetic origin of the strains. In particular, 11 variable positions of OmpA (*p* < 0.043), 32 of OmpC (*p* < 0.046) and 1 of OmpF (*p* < 0.001) were differentially distributed between groups of strains based on their phylogroup (Supplementary Table S6). In most cases, the amino acid variants were shared between A and B1 phylogroups and these were different from those of B2 and D strains, which in turn presented the same variant.

### OMPs Protein Expression

When growing in MH medium, no particular OMPs expression profiles were found for AIEC strains, although a slight increase in OmpC (AIEC: 92.9%; non-AIEC: 64.3%) and OmpF (AIEC: 70.4%; non-AIEC: 44.4%) prevalence was observed (Supplementary Figure S2). In general, OmpC expression was concomitant with OmpA and/or OmpF, but in the absence of OmpC, OmpF was always expressed together with OmpA. A group of strains showed only OmpA (7% of the AIEC and 22% of the non-AIEC).

### OMPs Gene Expression

RNA was extracted from two fractions of infected cell cultures: (i) from non-adhered/non-invading bacteria present in the supernatant (SN), and (ii) from bacteria adhering and/or invading the intestine-407 cells (A/I). RNA was thus obtained from mixed cultures (eukaryotic and prokaryotic); therefore, the greater amount of RNA was of eukaryotic origin in A/I fractions. In order to assess the differences in bacterial quantity between samples, the 16S rRNA was measured. As expected, considering that AIEC strains invade and survive intracellularly, 16S rRNA gene copy numbers varied according to pathotype in the A/I condition (*p* = 0.002), AIEC strains presented higher values (3.54 × 10^8^ ± 8.60 × 10^7^ 16S rRNA copies) than non-AIEC strains (9.93 × 10^7^ ± 2.12 × 10^7^ 16S rRNA copies). In the SN condition, minor differences in 16S rRNA gene copy numbers were identified (AIEC 1.24 × 10^8^ ± 6.68 × 10^7^ 16S rRNA copies; non-AIEC 3.54 × 10^8^ ± 8.87 × 10^7^ 16S rRNA copies; *p* = 0.043).

OMPs gene expression was similar between AIEC and non-AIEC strains in the two fractions. In the SN fraction the log10RTA values were: *ompA* AIEC −0.239 ± 0.147, non-AIEC −0.330 ± 0.100, *p* = 0.345; *ompC* AIEC −0.274 ± 0.166, non-AIEC −0.272 ± 0.126, *p* = 0.940; *ompF* AIEC −0.146 ± 0.113, non-AIEC −0.244 ± 0.122, *p* = 0.732. In the A/I condition, while no differences were found between pathotypes for *ompA* and *ompF* (*ompA* AIEC 0.141 ± 0.042, non-AIEC 0.046 ± 0.062, *p* = 0.302; *ompF* AIEC 0.091 ± 0.085, non-AIEC 0.040 ± 0.094, *p* = 0.654), in *ompC* a tendency was perceived, being the non-AIEC strains the ones showing higher *ompC* expression (AIEC −0.166 ± 0.107, non-AIEC 0.043 ± 0.100, *p* = 0.086).

**FIGURE 3:**
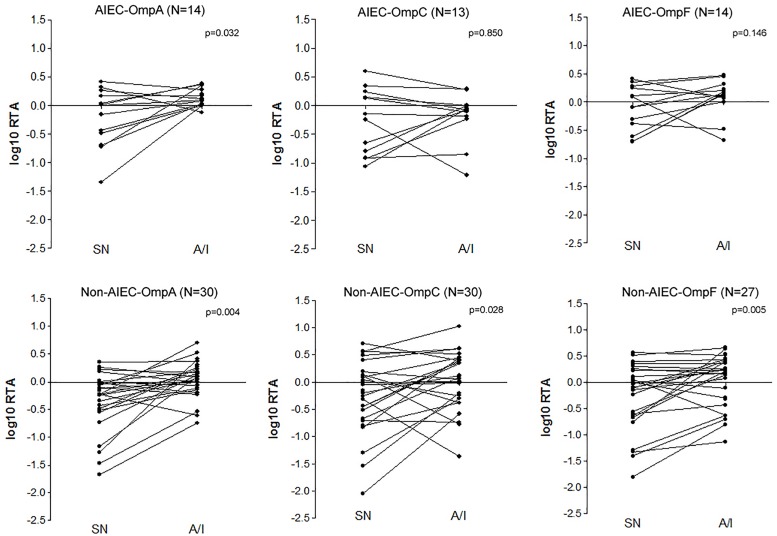
Paired tests evaluating the OMPs expression difference between supernatant and cell-associated fractions of infected I-407 cultures in each strain collection (AIEC and non-AIEC). Values indicate the logarithmic ratio of relative transcript abundance (RTA) of the target sample, being RTA = Efficiency ^(Ct target gene reference strain – Ct target gene sample)/Efficiency ^(Ct constitutive gene reference strain – Ct constitutive gene sample) (Pfaffl, 2001).

Despite no differences were found between pathotypes in each condition, paired tests were performed to uncover changes in OMPs expression for each group of strains according to the condition (SN or A/I) (Figure 3). AIEC strains showed significantly increased expression of *ompA* in the A/I fraction in comparison to the SN (*p* = 0.032) while *ompC* (*p* = 0.850) and *ompF* (*p* = 0.146) gene expression was similar in both conditions. All AIEC strains showed this *ompA* increase with the exception of AIEC04, AIEC06 and AIEC11. Conversely, although there is a high variability between samples, non-AIEC strains showed differences in gene expression between conditions for all of the three genes studied (*p* < 0.028), being in all cases higher in the A/I fraction in comparison with the SN fraction. Exceptionally four strains showed reduced expression of *ompA* (ECG01, ECG12, ECG21, and ECG28), seven of *ompC* (ECG01, ECG04, ECG12, ECG16, ECG43, ECG28, and ECG11) and six of *ompF* (ECG04, ECG12, ECG26, ECG28, ECG11, and K-12). These groups of strains presented no specific characteristics in terms of OMPs gene sequence, phylogroup, antibiotic resistance or virulence gene profile.

Despite there was much variability within each group, differences in gene expression were seen according to the phylogenetic origin of the strains, for *ompC* expression in both conditions (*p* < 0.023) (Supplementary Table S7). In this case, gene expression of strains from B2 phylogroup was lower than those of B1 or D phylogroup (*p* < 0.034). In terms of *ompA* and *ompF* expression, no differences were seen regarding strain phylogroup (*p* > 0.249) (Supplementary Table S7).

## Discussion

In gram-negative bacteria, OMPs have been shown to contribute not only to the structural integrity of the outer membrane, passive ion and solute transport but also to stress survival, bacterial virulence and resistance to antibiotics (Hancock et al., 1987; Torres and Kaper, 2003; Smith et al., 2007; Martínez-Martínez, 2008; Mittal et al., 2011; Liu et al., 2012; Hejair et al., 2017). Given that amino acid substitutions in these genes or differences in their expression have been implicated in the adhesion and invasion capacities of some *E. coli* pathotypes (Torres and Kaper, 2003; Rolhion et al., 2007, 2010; Maruvada and Kim, 2011; Mittal et al., 2011; Liu et al., 2012), in this work we sought to investigate the distribution of OMPs amino acid substitutions in a collection of AIEC and non-AIEC strains, along with the differential OMP expression of those strains in two distinct conditions (growing in suspension in a cell-culture medium and adhering/invading IECs), to define whether these proteins can contribute to AIEC virulence.

Little evidence exists on the putative role of OMPs in AIEC virulence (Rolhion et al., 2007, 2010). It has been suggested that OmpA interacts with the overexpressed receptor Gp96 of the intestinal epithelium of CD patients to promote AIEC invasion (Rolhion et al., 2010). The authors found five OmpA amino acid variants (V114I, F131V, D132Y, T228N, and A276G) in AIEC LF82 relative to *E. coli* K-12, that could be responsible for the increased invasion ability (Rolhion et al., 2010). Nonetheless, these amino acid positions were not conserved in the majority of our AIEC strains and did not correlate with the adhesion and invasion abilities of the strains. Moreover, LF82 OmpA variant was found in non-AIEC strains. In agreement with a previous study (Miquel et al., 2010), we found that OmpA gene variants were similar between AIEC, IPEC, ExPEC, and non-AIEC strains. This suggested that the five amino acid variant positions previously described are not relevant in our strain collection. Although no particular substitutions located in the *N-*terminal of OmpA were found to be associated with AIEC, an amino acid substitution located in a periplasmic position (A200V) was associated with pathotype and even bacterial adhesion. Because modifications in the periplasmic site of the protein may lead to misfolding of extracellular loops and thus also compromise protein-receptor interactions (Wang et al., 2016), functional studies could be conducted to assess the effect of this amino acid substitution in the AIEC phenotype.

The role of *ompC* expression in the interaction of AIEC with IECs under conditions of high osmolarity has been previously analyzed by Rolhion et al. (2007). Although reduced adhesion and invasion levels were reported in the LF82 OmpC-mutant, the wild-type LF82 phenotype was restored by overexpressing the RpoE (σ^*E*^) regulatory pathway in a LF82 mutant that did not express OmpC. Therefore, the authors concluded that OmpC involvement in the ability of LF82 to adhere to and invade IECs was indirect (Rolhion et al., 2007). To our knowledge, only two studies have examined OmpC prevalence in AIEC strains (Miquel et al., 2010; Prorok-Hamon et al., 2014), and this is the first work analyzing the OmpC sequence in more than one AIEC strain. Our results showed that this gene is widely present among *E. coli* strains regardless of the pathotype, as previously postulated (Miquel et al., 2010; Prorok-Hamon et al., 2014). Moreover, four amino acid substitutions were differentially distributed among AIEC/non-AIEC strains (S89N, V220I, and W231D) or associated with increased adhesion and/or invasion capacities (V220I and D232A), with one of the substitutions located in the extracellular region of the protein (D232A). Notably, it has been previously suggested that variations in the extracellular residues of OmpC may influence bacterial virulence because reduced adherence to macrophages has been reported in *Salmonella typhimurium* with an altered extracellular OmpC region (Negm and Pistole, 1999).

To date, studies examining OmpF amino acid substitutions have focused on analyzing the implication in antibiotic resistance (Benson et al., 1988; Zhang and Ferenci, 1999; Ziervogel and Roux, 2013), but information on the role of OmpF in bacterial pathogenicity remains poorly understood. Nonetheless, a role for OmpF has been pointed out in avian pathogenic *E. coli*, this protein is involved in adhesion and invasion to mouse brain microvascular endothelial cells *in vitro* and in brain, blood and lung colonization *in vivo* (Hejair et al., 2017). Currently, no evidence exists on the sequence variance of the *ompF* gene in AIEC, and its expression has been only studied in the AIEC LF82 strain (Rolhion et al., 2007). Here, we found no specific OmpF sequence variant associated with AIEC, however, two residues present in the extracellular (E51 and M60) domain correlated with high adhesion capacity of the strains.

Taken together, these data demonstrate that OMPs gene sequence variants are not sufficient to predict the AIEC pathotype due to their low sensitivity and specificity. However, the data contribute to increasing knowledge on AIEC genetics. Moreover, although new putative pathoadaptative mutations that may influence adhesion and/or invasion of strains have been presented, isogenic mutants to confirm its implication in AIEC virulence are required. Differences in the OMP protein sequence might be explained by the origin of the strains, as most of the variable positions were linked with the phylogroup, which is consistent with the observations of previous studies where sequence variations of other AIEC-associated virulence genes, for instance FimH, were investigated (Iebba et al., 2012; Dreux et al., 2013).

In an attempt to discriminate the AIEC pathotype among commensal *E. coli* isolates, OMPs protein expression profile was examined using overnight cultures. However, no differences were found. Thus, an analysis of OMPs expression during infection was carried out since external signals might be necessary to induce the expression of virulence factors.

This is the first study analyzing differential gene expression between a collection of AIEC and non-AIEC strains during IECs infection. Non-AIEC strains showed higher *ompA, ompC*, and *ompF* expression in the A/I fraction than in the SN of infected cultures, while AIEC strains only presented differences between conditions for *ompA* gene expression. According to the methodology applied in this study, the A/I fraction includes all bacteria that are in contact with the epithelial cells, both the adhered and the intracellular ones. Then, one may suspect that non-AIEC strains in the A/I fraction correspond to adhered bacteria and that in the case of AIEC correspond to both adhered and intracellular bacteria. Therefore, in the non-AIEC context, we can hypothesize that increased OMPs expression could be related to the adhesion of bacteria to IECs. Similarly, in the AIEC context, in concordance with the observations Rolhion et al. (2010), increased expression of *ompA* could also be related to adhesion. In contrast, *ompF* and *ompC* gene expression may be reduced in the intracellular AIEC bacteria in order to be protected from the acidic pH and from passive diffusion of oxidative residues, proteolytic molecules and antimicrobial peptides encountered in lysosomes and other solutes that might be created inside the eukaryotic cell through the OMPs channels. This hypothesis is supported by previous works, such as Lucchini et al. (2005) who reported reduced *ompC* and *ompF* expression levels during epithelial infection by *S. flexneri*, as well as other studies that suggest that OmpC and OmpF are required for low pH survival (Sato et al., 2000; Sainz et al., 2005).

Since OMPs gene expression may be regulated by, for example, the two-component OmpR-EnvZ regulatory system (Rolhion et al., 2007), the expression of other virulence factors could also be influenced by the same regulatory pathways. Therefore, it would be interesting to focus future studies not only on OMPs expression but also on coregulated genes. Moreover, it is unclear whether the gene expression reported in the A/I fraction was due to the adhered or the intracellular bacteria because it included all bacteria that were in contact with IECs. Therefore, additional studies differentiating between both fractions would be of interest in order to decipher whether gene expression levels observed are due to the intracellular bacteria or just an adaptation of the bacteria before entering the cells.

Our work provides new insights regarding OMPs sequence and expression in a wide collection of AIEC strains and adds knowledge about AIEC gene expression during IECs infection. We conclude that, although particular mutations in *ompA*, *ompC*, and *ompF* gene sequences may enhance the adhesion and invasion capacity of AIEC strains, they are not crucial for the adherent-invasive phenotype. Notwithstanding, differential gene expression of these OMPs during infection may contribute to AIEC pathogenicity by enhancing IECs adherence and intracellular persistence. Future studies looking at OMPs gene expression in other pathotypes should be conducted to determine whether it is a specific AIEC trait.

## Data Availability

The raw data supporting the conclusions of this manuscript will be made available by the authors, without undue reservation, to any qualified researcher.

## Author Contributions

MM-M, CC-F, and LM-M designed the study. CC-F, SB, and BR obtained the data. CC-F and SB performed the statistical analysis. CC-F and MM-M drafted the manuscript. SB, BR, and LM-M revised the manuscript. MM-M obtained funding.

## Conflict of Interest Statement

The authors declare that the research was conducted in the absence of any commercial or financial relationships that could be construed as a potential conflict of interest.
